# A Dialogue in the Medical Perspective—Body Mass and Nutritional Status Disorders during the Development Period

**DOI:** 10.3390/children9091360

**Published:** 2022-09-06

**Authors:** Karolina Kozioł, Beata Kazek, Dorota Sikora, Anna Brzóska, Justyna Paprocka, Ewa Emich-Widera

**Affiliations:** 1 Department of Pediatric Neurology, Faculty of Medical Sciences, Medical University of Silesia, 40-752 Katowice, Poland; 2 Department of Pediatrics and Pediatric Endocrinology, Faculty of Medical Sciences, Medical University of Silesia, 40-752 Katowice, Poland; 3 Child Development Support Center “Persevere”, 40-583 Katowice, Poland; 4 Specialty Diagnostics and Therapy Services, 43-200 Pszczyna, Poland

**Keywords:** nutritional status, eating disorders, neurodevelopmental disorders

## Abstract

Eating disorders among children and youth are a serious social problem. The time of development is the starting point in shaping eating patterns. Proper nutrition provides the basis for psychophysical development. A knowledgeable pediatrician can improve society’s health by engaging parents and, later, the child or youth. We offer knowledge on the nutrition basics and the commonly available tools to assess the nutritional status. We will discuss the characteristics of eating and body mass disorders in developing children. We will provide information on the warning signals of eating and body mass disorders and recommend prophylaxis. The reader will be familiarized with the motivational dialogue as an effective control tool for the discussed health issues.

## 1. Introduction

Nutritional status is “a state of health that results from the usual consumption of food, the intake and the utilization of the nutritional ingredients and the pathological factors affecting these processes” [1]. The nutritional status is derivative of age, sex, the amount and the quality of the consumed food, physical activity, and social and economic factors.

Proper eating is the basis of proper development and growth. It also lowers the risk for cardiovascular diseases in the adult age. Health education is needed to be introduced among children and youth in order to reduce the occurrence of body mass disorders. Parents, school counselors, and doctors should educate the children and the patients about the necessity of a proper, balanced diet and promote an active lifestyle. The recipients of these recommendations should not be limited only to overweight people as they should also include slim people. This is because having the proper body weight during childhood, puberty, and youth does not mean that the development of overweight in later years will not occur [2].

The results of the studies and analyses conducted among the children and the youth in Poland indicate significant deficiencies in the nutritional state among nearly 25% of that population. Underweight has been indicated in 18% of the group of boys and 20% of the group of girls. The children and youth with overweight constitute around 13% and with obesity around 8% of the population; despite the growing awareness about the risk factors, the overweight problem continues to target even younger people.

To introduce the proper intervention and prevent the development of the illness, the observation and early detection of body weight disorders among children should be the obligation of not only the parents and the primary care physicians but also of the school counselors and the school nurses.

## 2. The Basics of the Children’s Nutritional Status

It is important to pay attention to the frequency and the ingredients of the meals consumed. The proper preparation of the meals provided to the child during the day is necessary for creating healthy dietary habits and eating behaviors [3].

Children should consume between four and five meals during the day, including the three basic meals: breakfast, lunch and dinner [4]. Pursuant to the dietary guidelines, the energy from carbohydrates should consist of 45 to 65% of the total energy intake. On the other hand, fats should supply 35 to 40% of the total energy intake to provide for the child’s energy expenditure, growth, and development [5]. The unsaturated fatty acids are important in the child’s diet as they are the essential components of the neuron cell membranes and retina [6]. The diet’s minimum amount of protein should not be less than 1 g/kg of the child’s body weight. The average requirement for protein in children is 5 to 15 g.

Moreover, the proper amount of iron in the diet is essential. Children aged 4–8 years need 10 milligrams of iron daily, while older children aged 9–13 need 8 milligrams. Teen boys should get 11 milligrams of iron a day, and teen girls should get 15 milligrams. Adolescence is a time of rapid growth, and teen girls need additional iron to replace what they lose monthly when they begin menstruating. Currently, the vegan or vegetarian diet is very popular and common in the pediatric population but unfortunately, poorly balanced can lead to iron deficiency and, consequently, anemia. Pollitt et al. found that children aged 3-6 years with iron deficiency or due to iron deficiency reach lower scores on Bayley Scale than access to Mental Development. After starting treatment with an iron preparation, amelioration was observed [7,8].

Although there are different dietary recommendations for vitamin D depending on the country, vitamin D deficiency can obviously provoke fatigue, osteomalacia or even development delay. An analysis of data from the 2015–2016 National Health and Nutrition Examination Survey (NHANES) found that the average daily vitamin D intakes from foods and beverages were 196 IU in children aged 2–19 years [9]. According to the Food and Nutrition Board, the recommended dietary allowance for vitamin D in children aged 1–18 years is 600 IU [10].

Monitoring the daily caloric intake allows for maintaining the proper body composition, which translates into health and optimal physical fitness. The energy balance is maintained when the intake of the energy derived from the consumed food equals the expenditure of energy. Unfortunately, body mass and eating disorders play a significant role in children. Pediatricians, teachers, and school nursers should pay attention to children’s diet. If they are exchanging food during the pauses? Are they eating only unhealthy fast food? Are they avoiding eating and hiding in the toilet? These are probable questions to consider. The authors are convinced that teaching the basics of healthy eating and a healthy lifestyle at school will result in a long healthy life in the future and proper development and nutrition habits in a pediatric population.

## 3. Pediatric Nutrition Risk Screening Tools

The following tools are used to screen nutritional risk in children:

(a) Screening Tool for the Assessment of Malnutrition in Pediatrics (STAMP)
(b) Screening Tool Risk on Nutritional status and Growth (STRONGkids),
(c) Paediatric Yorkhill Malnutrition Score (PYMS),
(d) Pediatric nutrition screening tool (PNST),
(e) Simple Pediatric Nutritional risk Screening tool (SPENS).

Nutritional risk screening is recommended by the European Society for Pediatric Gastroenterology, Hepatology and Nutrition (ESPHAGAN) and the American Society for Parenteral and Enteral Nutrition (ASPEN) for all hospitalized children. It allows distinguishing the group of children of special care during hospitalization. Thanks to early diagnosis and early intervention, patients have a chance to recover faster and complete treatment. It also reduces the risk of complications [11]. Child malnutrition leads to a greater risk of infection and, consequently, a higher risk of death. It is the single biggest contributor to under-five mortality. (UNICEF) Additionally, it can lead to social and mental retardation and growth deficiency. It is essential to mention also the influence on economics. The mental impairment caused by malnutrition is permanent and directly linked to productivity loss.

The Eating Attitudes Test (EAT-26) is used to assess nutritional attitudes [12].

Tools to help identify eating disorders:

- The Eating Disorders Examination, EDE,
- The Eating Disorder Examination Questionnaire, EDE-Q,
- The Eating Disturbances in Youth Questionnaire, EDY-Q,
- Eating Disorders Screening Tool (NEDA),
- Children’s Eating Disorder Examination-Questionnaire (ChEDE-Q8),
- Adolescent Binge Eating Questionnaire (ADO-BED),
- Eating Disorder Screen for Primary Care (ESP)

## 4. The Tools Allowing for the Assessment of Children’s Nutritional Status

In order to assess the children’s nutritional status, it is important to know the proper tools. The basic parameters necessary to assess the somatic state are the anthropometric measurements conducted and analyzed systematically. These parameters include:

(a) Body length and height

For a child that has not achieved an upright position, the length of the body should be measured. The length is measured in a lying position between the child’s tip of the head, placed in the nose-ear leveled position, and the soles of its feet. The body height is measured with a stadiometer for a child that has achieved an upright position [13]. The person being measured should be standing up straight with their back turned towards the stadiometer, ensuring that the head, the arms, the buttocks, and the heels are touching the ruler. The arms should be hanging down naturally at the child’s sides. The ear canal should be leveled with the chick bone [13]. The measurements should be rounded to the nearest tenth.

(b) Body mass

The body weight measurement is conducted using the standard infant weighing scales. The newborn or an infant should be placed or seated on the scale without any clothing or a diaper. The measurements should be rounded to the nearest 10 g [13]. For an older, walking child, the measurement should be conducted in an upright position using a medical weighing scale. The child should be standing on the scale in underwear. The measurement should be rounded to the nearest 100 g [13]. The measurements of the body weight should be conducted regularly; in the first half of the first year of life, every month, in the second half of the first year, every three months, throughout the following three years of life, twice a year, and in later years, once a year [14]. It is worth adding that the birth weight not only determines the morbidity and mortality of the newborns but also translates into adult life. Low birth weight and heavy birth weight increase the risk of developing obesity and type 2 diabetes in adult life [15,16].

(c) Skinfold thickness

The next indicator of the nutritional state is the skinfold thickness. The measurements are conducted in three places: the abdomen (around the umbilicus), above the triceps brachii muscle (in the middle of its length), and on the back (underneath the inferior angle of the scapula). The thickness of the skin fold is measured on the left side of the body. The examiner should take a fold of skin to lift a layer of the skin and the fat tissue, leaving out the muscle. The caliper should be applied 1 cm distal from the marked site, at the right angle, and the result should be provided in millimeters approximately 2 s later. The analysis requires two measurements to be considered [17]. The total of 3 skinfold measurements will indicate the thickness of the subcutaneous fat. Based on the above measurements, the circumference of the arm muscle can be measured using the following formula: mid-upper arm circumference = [circumference of the arm muscle-(3.14 × the thickness of the arm skinfold)]. The result is the indicator of muscle mass [13].

(d) Waist circumference

The waist circumference measurement is conducted using the anthropometric tape measure placed horizontally in the middle between the highest point of the iliac crest of the hip bone and the lowest part of the costal margin in the midaxillary line. The abdominal muscles should be relaxed. In clinical practice, this measurement is conducted to indicate the content of the intra-abdominal fat [13,18].

(e) Hip circumference

The hip circumference should be measured by passing the measure horizontally at the highest point of the buttocks muscles below the ilia [18].

(f) Waist Hip Ratio (WHR)

The waist-hip ratio is the indicator of fat tissue distribution. It is calculated by dividing waist circumference measured in centimeters by hip circumference measured in centimeters. The results obtained for women, which are equal to or greater than 0.85, indicate intra-abdominal (visceral) fat obesity, and the results, which are less than 0.85, indicate femoral-gluteal obesity (gynoid, peripheral). On the other hand, for the results obtained for men, the cut-off point for this indicator is 0.9 and 2.6, respectively [18].

(g) Waist to Height Ratio (WHtR)

The waist-to-height ratio is an indicator of fat tissue distribution. It is calculated by dividing waist circumference by height in centimeters. The normal value is below 0.5. Higher values indicate a higher risk for cardiovascular diseases or diabetes. The WHtR index, similarly to the waist circumference, highly correlates with the content of the abdominal fat tissue, measured with imaging methods [13,15]. It is a better indicator than BMI in assessing the risk related to obesity and metabolic syndromes. It is also a better indicator of the risk of morbidity and mortality [19].

It is indispensable to translate the abovementioned anthropometric measures to the biological reference systems when assessing children’s nutritional status, indicating whether the particular anthropometric measure stays within the normal range or exceeds it. The most popular reference systems will be presented in the following paragraphs, which are routinely utilized in pediatric practice.

(a) Growth charts

The most important reference systems are the growth charts. A percentile indicates the percentage of the frequency of a certain characteristic manifesting in the studied group. It divides the value of the parameters of the studied population into 100 parts. For example, if the value of the studied characteristic, height, is in the 50th percentile, it means that it is the average height for children of the same chronological age. If the result were marked in the 40th percentile, it would mean that 60% of the children of the same chronological age are taller, and 40% of the peers are shorter or the same height.

The growth charts are graphic illustrations of the location of the studied characteristic and the changes in its percentile value within a certain period of the child’s development. Plotting the points which refer to the particular percentiles in the chart with a line will result in obtaining the percentile curves which reflect the development trajectory of the examined characteristic. In order for such a percentile position to be deemed correct, it cannot exceed the normal range of more than two percentile curves. A significant deviation from the range indicates the need for further diagnosis. That is why the regularity of recording the measurements in the growth charts is of great importance so that the development tendency may be identified.

According to the WHO norms, the 2nd and the 98th percentile are cut-off values of the normal range; 99.5% of the population is within this range. The range between the 25th and the 75th percentile is considered the narrow normal range, and the range between the 10th and the 90th percentile is considered the broad normal range. What requires the attention of the pediatrician, caregivers, and school counselors is the values below the 2nd and the 98th percentile [20].

It is interesting to mention that the WHO Child Growth Standards for children between the ages of 0–5 are applicable worldwide. It has been indicated that the growth of children in this age group who are living on different continents is in close proximity if certain basic biological needs, such as breastfeeding, are ensured. That is why it is a tool that may be utilized to assess the development of newborns and young children across different countries and ethnic groups.

Based on the growth charts, it is possible to estimate the child’s height at the age of 18 with high probability, provided that the child’s measurements remain within the same percentile range and both parents’ growth potential is known. Parents often ask about how much their children should weigh. It is important to remember that body mass is always assessed in relation to height. The body mass is the value of the 50th percentile of the norm in relation to the particular height.

(b) Numerical tables

The numerical tables are more simple yet less popular reference systems. They provide the arithmetic means and standard deviations of the particular characteristic in relation to the particular age and sex. The norm is marked by the value: average x_1 SD (standard deviation).

When assessing the child’s nutritional status, it is important to mention the very popular tool, the body mass index (BMI). BMI is calculated in the following way: BMI = body mass (kg)/body height (m^2^). The BMI index informs us about the deviated weight/height proportions. The child’s calculated BMI value must be compared with the norms presented in the growth charts [21]. The values below the 2nd percentile indicate body mass deficiency, and the values above the 98th percentile indicate obesity [22]. Nevertheless, it is important to remember that this indicator is not perfect. It does not provide information on the distribution of the fat tissue and the physiological fluctuations in the proportions of the fat mass, the muscle mass, and the bone mass, which depends on the type of the body frame. Sex, age or the type of body frame and fitness are not considered when calculating BMI [17]. A bodybuilder whose BMI would indicate extreme obesity due to the developed muscle mass may serve as an example of that as the fat mass of such a person is very low.

Another, more advanced method of assessing the nutritional state comes to aid being the analysis of the body composition in which the bioelectrical impendence measuring devices are utilized. Electrical resistance is used in this method. Fat tissue and extracellular fluid show active electrical resistance, resulting in lower electrical conductance. The tissue with high water content shows capacitive resistance [23].

The test takes a few minutes. The patient should stand on the scale with electrodes. Next, a small electrical current of 0.8–1 mA is run through the body, which is practically not detectable, and then the bioimpedance is measured. The device cooperates with a computer program that conducts the calculations. The muscle mass, the visceral tissue, the entire content of water in the body, the bone mass, the daily energy requirement (in kcal or kJ), the metabolic age and the body mass are measured during this test. The increased percentage of fat tissue in the body informs about the increased risk of health problems [23].

The measurement should be conducted on an empty stomach or at least 4 h after the last meal. In order to obtain a reliable result, the height of the examined person should be measured carefully. If the analyzer does not have a weight scale built-in, the patient should also be carefully weighed [23].

No harmful effects have been identified in using this method. The availability and the simplicity of this method of measuring bioimpedance helped to make its way into diagnosing and treating obesity in children and adults [23,24]. As perplexing as it may sound, obese people are undernourished at the cellular level. Even though the food is consumed in excessive portions, it is not of good quality. This has been proven by the body composition analysis, which shows a high percentage of fat tissue, increased visceral mass, and low bone density.

To summarize, the discussed anthropometric measurements, the biological reference systems, and the analysis of body composition obtained by the bioelectric impendence method are fast, inexpensive, and simple methods of assessing the nutritional status of children [23,24]. The anthropometric method of assessing body composition is generally available and noninvasive to determine the child’s total body fat and muscle mass at any age. It is also possible to reference these measurements to the national physical development standards. The disadvantage is that there is a greater risk of an observational error occurring in this method more often than in other methods.

The bioelectric impedance method also meets the simplicity and safety criteria and is even more reliable than the anthropometric method. The limitations include the only available foreign standards, where the tests may be conducted not earlier than at the age that allows for cooperation, and that is usually the age of 5 [24].

In order to optimize the presented method of the assessment of the nutritional status, both methods, the detailed anthropometric measurements and the body composition analysis would need to be utilized.

## 5. The Characteristics of the Body Mass Disorder in Children

In order to determine the cause of the child’s body mass disorder, it is necessary to conduct a careful interview, perform a physical examination, and assess the nutritional status using the methods described above. The most frequent causes and effects of body mass disorders in children are described in the following paragraphs.

(a) Underweight

The loss of body mass may result from illnesses of almost all of the systems and organs. It is an ambiguous symptom that may signify a chronic disease [25]. The most frequent cause of weight deficiency in children is an incorrectly, in terms of quality and quantity, balanced diet. The causes may include premature birth, high-risk pregnancy, placental insufficiency, and the use of stimulants by the mother (nicotine, alcohol). Sucking disorders, birth defects, or lack of food are among the causes related to infancy [26]. The next group of causes of undernourishment among children includes the esophagus disorders, such as celiac disease, acid reflux disease, ulcer disease, food allergies and intolerance or inflammatory bowel disease. Other causes include chronic diseases, such as heart and cardiovascular defects, defects of the respiratory system, chronic respiratory failure, chronic kidney disease, cancer, and endocrine disorders, including diabetes, genetic disorders, infections, and neurological diseases. Let us not omit the psychological and psychosocial disorders and the iatrogenic diseases induced by drugs and hospitalization. Still, the family and the constitutional factors are as important as the diseases.

The results of body mass deficiency can include decreased physical fitness and immunity, hair loss, psychomotor delays, decreased strength, and hypotonia [25]. The use of the restrictive diet may cause nutritional deficiency, especially in the area of vitamin and iron content, which may result in anemia. During puberty, the proper amount of the fat tissue is required to ensure the development of the reproductive system. Insufficient fat tissue may cause menstrual disorders. Undernourishment also increases the risk for a psychological disorder and may cause irritability, impaired concentration and difficulty sleeping. After the cause of the body mass deficiency is determined, the proper treatment must be introduced, as chronic undernourishment affects the child’s development and may impair the child.

(b) Overweight and obesity

During a child’s development, the environmental factors, including incorrect eating habits in the family, emotional problems, stress, limited physical activity, as well as the incorrect diet applied by the pregnant woman, play a significant role in the development of obesity. The most frequent cause of overweight and obesity is excess energy intake which is caused by the excess energy consumed in relation to the energy expenditure for the basic metabolism, movement and thermogenesis. The other causes of secondary obesity are endocrine system diseases, such as Cushing’s disease, hypothyroidism, nervous system diseases, genetic disorders, and chronic steroid therapy [27].

The consequences of obesity include pulmonary disorders (bronchial asthma, exercise intolerance, sleep apnea), orthopedic problems (valgus knee, posture deficiency), as well as endocrine disorders (premature sexual maturation), fatty liver, arterial hypertension, strokes and atherosclerosis in the adult life [27]. The comorbidity of dermatological disorders and hypercholesterolemia is also frequent. The excess body mass may also lead to carbohydrate metabolism disorders, including metabolic syndrome [2]. The risk of children with excess body mass developing diabetes is 10 times greater than that of slim children [28]. We also need to remember the psychological consequences. Obese children are discriminated against by their peers; they develop low self-esteem and display an array of symptoms of depression that correlate with BMI [29]. Not being able to cope with social pressure, they choose the incorrect food products, contributing to weight gain, and that is how the wheel turns full circle. Furthermore, this leads to eating disorders [30].

## 6. Characteristics of Eating Disorders in Developing Children

Eating disorders are serious, often chronic and debilitating syndromes that usually commence during child development. It is especially important for parents and school counselors to stay vigilant and not miss disturbing symptoms. Although these disorders occur among females more often, it is also important to observe males. The classification of eating disorders is described in the newest fifth edition of the American Psychological Association’s Diagnostic and Statistical Manual of Mental Disorders, DSM-V [31]. Next, we will provide the characteristics of eating disorders in children and youth.

(a) Anorexia Nervosa

Anorexia nervosa is the most common disorder among developing girls. It is characterized by body mass deficiency—according to the International Classification of Diseases, it is in ICD-11 <5 percentile for children [32]. Furthermore, it is connected to the excessive desire to decrease body mass and undertake steps, such as restricting food intake or exercising intensely, to achieve that goal. Consequently, children are at serious risk of health problems, including sexual and reproductive health. Due to hormonal imbalance, the normal process of puberty is delayed. The development of the disease is often preceded by a change in lifestyle to the so-called healthy lifestyle, for example, introducing a vegetarian diet, avoiding high-fat food, and increasing physical activity (even in 30–80%) [33]. Exercises, just like food consumption, may take the form of rituals and disturbing that may cause frustration and aggravation in the patients [34]. Disturbed body image is also a symptom, where the entire body or certain parts of the body seem to be too fat [35]. Caregivers must be aware that anorexia carries the risk of death. The causes of death usually include somatic complications, cachexia or suicide [36]. The intervention undertaken in the early stage of the development of the disease has been proven effective [37]. That is why awareness of the symptoms and early intervention is so important.

(b) Bulimia Nervosa

Bulimia nervosa is a disease that is characterized by alternating episodes of binge eating and compensating behaviors. The latter may include self-induced vomiting, taking drugs to induce purgation or fasting. Binge eating is a way of controlling emotions, providing temporary relief, and decreasing the feeling of discomfort [38]. After such episodes, the increased feeling of shame and fear of gaining weight takes place. The mechanism of a vicious circle is at play here. The methods for decreasing body mass are the triggering factors for binge eating, and the patient decides to apply drastic measures again to burn the consumed calories and avoid gaining weight. Bulimia causes serious health and psychosocial consequences. Persons with this disease usually have an adequate body mass or are overweight, which makes the people from the immediate environment unaware of the problem they suffer from. Meanwhile, bulimia may lead to many health problems and death.

(c) Binge Eating Disorder

It is the most common eating disorder. It is characterized by the occurrence of binge eating with a feeling of no control over it at least once a week. These episodes are characterized by fast eating, eating without the feeling of hunger, and the continuation of eating despite feeling full. Eating often takes place in solitude, with the feeling of disgust at oneself and the feeling of guilt after such episodes. Stress, the problem with accepting own body mass, and the desire to adhere to a restrictive diet are considered to be among the possible factors triggering binge eating episodes [33]. It is worth remembering that only 12% of teenagers with this disorder seek out professional help, and it is important to observe children and youth in that respect as well [39].

(d) Avoidant/Restrictive Food Disorder (ARFID)

This disorder, characterized by avoiding or limiting food consumption, differs from the disorders described above in that the distorted body image or the fear of gaining weight does not occur here. Still, the health and the socio-emotional consequences can be just as serious. ARFID is connected with at least one listed symptom: a significant loss of body mass and/or growth deficiency, serious malnutrition, the necessity for oral supplementation or enteral feeding, and socio-emotional problems. Avoiding or limiting food consumption is characteristic of ARFID. It is caused by the sensitivity to the sensory characteristics of food products (such as the smell and texture of food), the fear of the unpleasant consequences of consuming food (chocking, gastric pain, etc.) or lack of interest in food (decreased appetite, lack or decreased feeling of hunger) [40,41].

Children and youth who suffer from ARFID usually have a minimal repertoire of the preferred food products (from a few to just over 10). Underweight, body mass that is adequate for the particular age and height, and overweight, depending on the caloric value of the preferred food, are observed in such children. They usually avoid social situations related to consuming food, such as special events or eating at the school cafeteria. It is significant for children and youth suffering from (ARFID) that in case their preferred food is not available, they choose hunger, which only worsens their health issues. It is important then to ensure that they have constant access to the preferred food and to introduce multidisciplinary care [40].

(e) Orthorexia Nervosa

Although this disorder is not included in the DMS—V classification of diseases, it is worth mentioning. It is characterized by excessive attention paid to healthy eating rules. Persons who are suffering from this disorder impose increasingly restrictive diets on themselves. They plan their entire days around preparing and consuming food. This becomes especially dramatic when these restrictive eating rules are applied to small children by the parents. Such an irrational diet may result in malnutrition which, in extreme cases, may even lead to death [42]. It is important to say that the role of school counselors is especially vital in reacting fast and alerting the proper authorities at the signs of such forms of domestic abuse.

## 7. Warning Signs of Body Mass and Eating Disorders in Children

Noticing the warning signs of eating disorders allows for the application of the proper procedures to limit the serious health consequences. Obviously, an unusually low or high BMI or an unusually light or heavy weight in proportion to height, as well as a sudden weight loss, should alarm the caregivers. These symptoms are caused by the use of restrictive diets or a change in eating habits. It is important to pay special attention to socially withdrawn children, especially in food consumption, i.e., such as during breakfast breaks or school field trips. Comorbidity of other psychological disorders is observed in 45–97% of children with body mass and eating disorders. The most common disorders include anxiety and mood disorders, obsessive-compulsive disorder, substance abuse and personality disorders [33]. Another warning signal is excessive attention paid to the body mass and the body image, as well as the distorted perception of the body’s shape. The latter does not need to refer only to those children with low weight but also to children suffering from overweight and obesity [43]. There may be difficulties with controlling chronic illnesses that require the application of special diets, such as diabetes or celiac disease. Caregivers should also pay attention to disrupted menstrual cycles, heart arrhythmia, dizziness, fainting and pale skin. In such cases, medical diagnostic consultation is required.

The next group of warning signals includes compensating behaviors, such as abusing the drugs to induce purgation or weight loss pills, self-induced vomiting or excessive exercising. Abdominal pains, especially in connection with vomiting, may also be the first symptoms of an eating disorder. Thus, unusual tooth damage, dental enamel hypoplasia, and advanced tooth decay should alert caregivers and school nurses. On the other hand, physical education teachers should pay attention to the special involvement in physical activities with high risk for eating disorders, such as professional sports, dance or modeling. Moreover, growth deficiency and delayed puberty in children and youth should prompt diagnostic testing on body mass and eating disorders in children.

## 8. Shaping of the Nutritional Behavior, Habits and Preferences

Nutritional behavior is defined as complex actions undertaken to satisfy human beings’ needs, including choosing, storing, preparing, cooking and consuming nutritional products. Nutritional habits, which constitute acquired, learned, and repeated behavior, are used for nutritional, social and emotional purposes. The nutritional preferences portray either the positive or negative approach to the particular products. The whole process of shaping nutritional behavior, habits and preferences is complex and multiphase. The philosophy of appetite, hunger and satiety is the foundation of this process and is portrayed in Blundell’s satiety cascade. Hunger has been defined as the philosophical need for food in contrast to appetite, which has a psychological background, and satiety, which is the feeling of satisfaction. The satiety cascade shows four phases of the food flow, starting from appetite and hunger to the final satiety: the sensory phase, the cognitive phase, the pre-absorption phase, and the last post-absorption phase that involves the senses, several hormones and neurotransmitters. The other components may be grouped based on the areas they belong to, such as the family area, the individual area, the nutritional area and the social, demographic, and economic area (Table 1) [44].

The critical times to shape the proper nutritional behavior, habits and preferences are between the 13th and the 36th month of life. It is the time during which the main aspect of the child’s functioning is its curiosity that is also directed towards eating and the involved rituals. The child learns the food products with all its senses. Nevertheless, it is also a time of considerable criticism of everything that is new and unknown. Consequently, it is related to the risk of developing the transitional state of food neophobia. The role of the parents should be to show patience and consistency in presenting the child with new foods, tastes and fragrances as the final acceptance of the “novelties” may, according to scientific research, require even 10–15 trials. During this “absorbing” time, the most important thing is to present the child with healthy behavior related to broadly understood eating while creating a calm and pleasant atmosphere through the example set by the family [44,45,46,47].

The preschool time between the 3rd and the 6th year of life is especially significant due to the high psychomotor activity and increased need for macro- and microelements in proper proportions, along with the developmental potential in the form of the flexibility of the central nervous system directed to the cerebral cortex. The intellectual, motor, and social development during preschool time also translate into the nutritional development of the child, its nutritional behavior, habits and preferences that the parents and the preschool education influence. During this time, the child copies the nutritional behavior of the family members at home with greater awareness than previously, paying special attention to the place of consuming meals, the accompanying sounds and illumination, the time of day, the regularity, the type of food, the food products on the plate, their quality and quantity, and their proportions in relation to each other [45].

Children of school age are characterized by the educational approach to the problem of food that includes the school environment in shaping nutritional behavior and preferences. The peer environment also plays an important role.

During the teenage period that overlaps with puberty, the awareness of own bodily appearance and attractiveness is increased. It becomes the next step in the conscious shaping of the young person’s behavior [44].

During each phase of the development, “the skeleton” of healthy eating, and the shaping of the related behavior, habits and preferences, constitute the basic rules of the proper feeding and consuming meals that involve the child and its family.

## 9. Prevention of Body Mass and Eating Disorders in Children

The prevention of body mass and eating disorders among the children and youth population requires a multidisciplinary approach to consider the child, their family, and their environment—preschool and school. That is why the role of the school counselors and the teachers and their awareness of these problems is so important. The purposes of the primary prevention practice must shape the correct eating behaviors, which are instilled by the family food environment. We have already mentioned the nutritional basics, including breastfeeding up to the 6th month of the child’s life. It allows the child to learn about the different taste stimuli, familiarize themselves with them, and shape their abilities to recognize the feelings of fullness and hunger. The parents are responsible for learning about diverse and healthy foods and promoting different food products. Still, the child should eventually be able to decide what and how much of the given food it will consume. The method of repetitive exposure (6 to 15 repetitions) is the best method of facilitating familiarization and increasing the acceptance of new food products [48,49]. It is, therefore, not recommended to quit exposing the child to particular food products too fast. It is also worth ensuring a variety of food products to avoid the child getting bored and losing interest in a particular food [50]. While shaping the correct eating habits, teaching the child that food is not a form of reward or punishment is necessary.

The immediate environment (parents, grandparents, teachers, peers and other authorities) models the child’s eating preferences and habits, which is why it is so important to draw adults’ attention to their own eating beliefs and the resulting eating behavior. Often, parents and grandparents do not have sufficient knowledge about the proper nutrition of children, resulting in various difficulties (including improper eating style, malnutrition, overweight or obesity, diabetes, and eating disorders). The style of feeding parents and the environment is also of great importance. Pressure and excessive control block building a child’s positive relationships with food and may also aggravate the child’s difficulties in this area. Excessive parental control of meals (in terms of the amount of food eaten, the way it is consumed, and the duration of the meal) results in weaker self-regulation of the child in terms of the feeling of hunger and satiety [51].

A responsive approach to feeding assumes mindfulness and interest in eating, noticing and responding to internal signals of hunger and satiety, the ability of the child to communicate their needs to the caregiver, and the pursuit of independence in eating. The responsive parent tries to be flexible, cares about the food atmosphere and the consistency of meals, responds adequately to the signals of hunger and satiety sent by the child, adjusts his expectations to his needs and abilities, and supports them emotionally [45].

Research indicates the role of observational learning, with particular emphasis on the role of parental attitudes and behaviors. Children’s food preferences change by watching others eat (including parents, grandparents and peers). Research suggests that children observe not only their parents’ food consumption but also their relationship to food and their relationship to their own bodies [52]. Media creations, including advertisements and websites [50], the impact of which can be both positive and negative, also play a significant role in shaping children’s nutritional behavior. Research shows that television exposure is of high importance in perpetuating the eating behavior that promotes obesity in children [53]. From year to year, more and more children suffer from overweight and obesity, which have health and psychosocial consequences [54].

It is important from an early age to develop nutritional knowledge and a child’s positive relationship with food because the best form of treating diseases is to prevent them through preventive measures [54]. Because the nutritional behavior of parents has a significant impact on the nutritional behavior of their children, it is necessary to educate not only the children but also the parents and teachers. This will allow for the adequate development and maintenance of health and good psychosocial functioning of the whole family [55].

School is becoming one of the key environments in which strategies to improve children’s eating habits should be developed. Such strategies include programs promoting proper nutrition, building positive relationships with food, and shaping nutritional knowledge [56]. USDA’s Food and Nutrition Service (FNS) operate child nutrition programs, which include: National School Lunch Program, the School Breakfast Program, the Summer Food Service Program, the Child and Adult Care Food Program and After-School Snacks and Meals [57].

During the school period, it is important to ensure that the child eats the meals at the table without watching TV or using the computer. It would be preferable for the meals to be consumed by the child together with the family. It is important to avoid having unhealthy food products, containing high levels of sugar, fat, or highly processed food at home. After the child returns home from school, the child should be given a fresh vegetable salad, yogurt, or fruit to eat. It is worth including children in the preparation process of healthy meals. It is important to pay special attention to youth eating under emotional distress, overeating, and self-induced vomiting. It is important to ensure that the child eats 3 to 5 meals at the same time each day (including breakfast). Caregivers should also talk to their children about what a healthy body really is. The indispensable element of body mass and eating disorders preventive practice is pro-health education in schools and the introduction of psychoeducation on building adequate self-esteem or coping with emotions [38]. The authors would like to emphasize the importance of the school teachers providing the information on the child’s behavior in the health reports in a diligent manner so that this information may serve as the basis for the doctor to be able to perform the proper diagnostic procedure and introduce the effective treatment.

## 10. Treatment of the Body Mass and Eating Disorders

Early intervention is of great importance in body mass and eating disorders because it provides a high probability of successful disease treatment. The child’s immediate environment, including family and school, should possess the proper knowledge of the eating-related problems and promptly react to the first signs of the first symptoms suggesting eating disorders. After detecting disturbing symptoms in students, the school counselors should take early intervention steps by collecting information from the other teachers and school nurses. The school counselor should plan the conversation with the student in a way that would provide the student with the feeling of safety and not the feeling of being attacked. It is worth including the school psychologist and offering the student to meet with the school psychologist in a comfortable environment. The student should be informed about the necessity of notifying the parents about the identified problem, as the teacher’s responsibility is to protect the student’s health and life [38]. The conversation with the parents cannot relate to the emotions of the student, her or his thoughts, or the information privately entrusted to the teacher. It is important to build a relationship with the student that is based on trust. The parents should be informed about the actions that would be undertaken in such situations, including psychiatric and psychological consultations at the psychological health clinic or a visit to the ER of a psychiatric hospital (in case the student does not want to cooperate and she or he is at the risk of a serious illness or death). The treatment of eating disorders is multidisciplinary in character—the patient’s somatic state should be treated, proper pharmacotherapy should be introduced, healthy eating patterns, and the different forms of psychotherapy (individual, family, and group) should be introduced. It is also important to provide the proper support for the student and the parents at the school [34].

Pediatricians, school nurses, teachers and specialists in various fields should be able to define the symptoms of eating disorders. Often their intervention depends on whether the child or adolescent and his parents receive adequate and tailored help. It is known that postponing diagnostic and therapeutic measures has a negative effect on treatment, making it difficult for the patient to recover [58]. Early intervention increases the chances of achieving the assumed goals, including the patient’s recovery.

Treatment of eating problems should be multidisciplinary and include the activities of various specialists, e.g., pediatricians, psychiatrists, gastrologist, psychologists, speech therapists, dieticians, physiotherapists and sensory integration therapists. The areas of activity of individual specialists are presented in Table 2.

## 11. A Dialogue in the Medical Perspective—A Motivational Dialogue

The question of whether the patient will want to change their behaviors would depend on the patient’s readiness phase. A person that is aware of the difficulties of the current situation and understands the estimated benefits of introducing changes will implement the suggested recommendations with a higher probability. An uncooperative patient requires the application of certain methods that will increase motivation [59]. One such method is a motivational dialogue performed during the consultations with the doctor, the counselor, and with the nutritionist, regardless of the level of the patient’s motivation level, which will result in permanent and long-term changes in behavior. The motivational dialogue is based on cooperation with the patient, respect for the patient’s decision making and autonomy, identifying the patient’s strengths, and responding to the patient with empathy. All of that aids the specialist in building trust with the patient [59,60]. Working with the unmotivated or ambivalent patient requires special involvement of the specialist, such as active listening and treating the patient as an equal or as an expert. Giving advice and automatic recommendations when the patient is not ready for changes may make them feel misunderstood and leave therapy [59]. When working with a teenager in the spirit of a motivational dialogue, we can increase the chance of instilling a cooperating approach in the patient which will bring progress in therapy. Young people have the need for acceptance and the need for consent to make decisions regarding themselves. It is a good idea to use open-ended questions, listen actively to the replies, and use every opportunity to boost the patient’s self-esteem when working with teenagers based on specific behaviors and resources. Being empathetic and responsive when conversing with young people is also important. It will encourage them to make the changes [59].

## Figures and Tables

**Table 1:** The components affecting nutritional behavior, habits and preferences.

Child	Sex, age, body mass index (BMI), prenatal stage, birth weight, eating during the newborn-infant stage and during childhood, early taste experiences, distribution of fat tissue, metabolism, physical activity, personality, knowledge on nutrition, ability to learn, state of health, behavioral pattern, control of health and emotions
Family	Sex, age, BMI, education, learned attitudes, knowledge on nutrition, food on the everyday menu, nutritional preferences, physical activity, nutritional habits, parenting style, accountability, connection with the child, meal size, access to healthy food products, the atmosphere at the table, style and time of day of consuming meals, regularity of consuming meals, sounds and illumination during eating, family and social relations, family model
Food	Taste, smell, look, texture, accessibility, energy value
Demographic, economic and social conditions	Family income, ethnic background, habits and culture, social media, high technology devices, nutritional programs in school, recreational programs and accessibility to such programs, neighborhood
Genetic predispositions	(1) Predisposition to accept sweet taste and lack of tolerance to accept a bitter and sour taste (2) Predisposition to lack the acceptance of new and unknown food products along with attachment to the familiar ones (3) Predisposition to associate particular food product with impressions and effects after eating it

**Table 2:** List of areas of activity in the diagnosis and treatment of eating problems.

Specialist	Areas of Activity in the Diagnosis and Treatment
Psychiatrist	- general mental state assessment, - differential diagnosis, - diagnosis of comorbidities, - implementation of pharmacotherapy in the case of comorbid disorders
Psychologist/psychotherapist	- mental state assessment, - assessment of comorbid emotional disorders, - conceptualization of the patient’s problem, - psychotherapy (adapted to the difficulties, needs and possibilities of the patient), - family therapy
Pediatrician	- monitoring height and weight according to the percentile graphs, - monitoring of micronutrient and macronutrient deficiencies, - supplementation, - diagnosis and treatment of comorbid medical problems
Gastrologist	- diagnosis and treatment of comorbid medical problems
Dietician	- analysis of the way of child’s feeding and eating, - evaluation of the child’s current 3-day menu, - nutritional needs assessment, - regulation of the meal and snack regimen throughout the day, - preparation of a meal plan adapted to the current needs and capabilities of the child, - monitoring food and fluid consumption, - supplementation
Speech Therapist	- examination of the condition of the articulation apparatus, - evaluation of biting, chewing and swallowing, - assessment of muscle tone in the oral area, - assessment of breathing patterns, - evaluation of oral reflex reactions
Physiotherapist	- assessment of motor development - rehabilitation adapted to the patient’s needs
Sensory Integration Therapist	- diagnosis of sensory disorders, - preparation of stages of desensitization therapy, - preparing recommendations for parents to work with their children at home

Source: own study based on Coglan, Otasowie 2019 and Bryant-Waugh et al. 2021 and own experience in working with patients with eating problems [58].

## Data Availability

Not applicable.
